# Surveillance of central-line-associated blood stream infections (CLABSI) in intensive care units of a tertiary care center in Western India

**DOI:** 10.3205/dgkh000618

**Published:** 2026-02-06

**Authors:** Prachi Verma, Shilpi Gupta, Sachin Bansal, Gagan Priya Pandey

**Affiliations:** 1 Mahatma Gandhi University of Medical Sciences and Technology, Jaipur, Rajasthan, India; 2 Mamta Medical College and Hospital, Bihar, India

**Keywords:** blood stream infections, central line-associated blood stream infections, CLABSI, central venous catheter, healthcare-associated infections, intensive care unit, India

## Abstract

**Introduction:**

Central-line—associated bloodstream infection (CLABSI) is the most prevalent type of healthcare-associated infection (HAI). It ranks as one of the primary contributors to both morbidity and mortality in hospitals globally. The occurrence of CLABSI is influenced by a variety of risk factors. It is essential to collect precise data on the trends of incidence rates, causes, and antimicrobial resistance patterns of central line-associated bloodstream infections (CLABSIs)in the setting to formulate empirical treatment regimens and emphasize robust infection control policy.

**Aims:**

To study the prevalence, etiology and antimicrobial susceptibility of CLABSI in adult medical and surgical ICUs in a tertiary care private hospital in Jaipur, India.

**Methods:**

This prospective observational study was conducted at the medical and surgical intensive care units for adults of Mahatma Gandhi medical college and hospital, Jaipur, Rajasthan, India from January 2023 to December 2023. The CLABSI event was identified according to the Centers for Disease Control (CDC) definition as part of the routine surveillance of HAIs. The incidence rates of CLABSI (per 1.000 central line days) were calculated. Identification and antibiotic susceptibility were determined using an automated blood-culture system.

**Results:**

During the study period, 59 patients experienced a single episode of CLABSI, with a mean prevalence rate of 15.6 per 1,000 central line days; the maximum rate was found in the neuro-ICU (21.6 per 1,000 central line days). The clinic-demographic profile showed 2.1–to-1 male:female ratio with maximum occurrence of cases in patients over 60 years of age (25/59; 42.4%). The most common site of CL insertion was the jugular vein (97%). The majority of cases (88.1%) occurred in patients with prolonged ICU stays exceeding 10 days with an overall mortality rate of 49.2% (29/59). The most common causative micro-organism was *Klebsiella pneumoniae* (17/59; 29%), followed by *Acinetobacter baumannii* (14/59; 24%). 83% (33/40) of Gram-negative isolates were resistant to carbapenem, an no isolates were resistant to colistin.

**Conclusion:**

The study revealed a disproportionately high incidence of CLABSI when compared with both developed and developing countries, with multi-drug-resistant Gram-negative bacteria being the predominant causative agents. Continuous surveillance, supported by infection control practices, e.g., hand hygiene and bundle compliance audits along with stringent antimicrobial stewardship, is vital in mitigating CLABSI.

## Zusammenfassung

**Einleitung:**

Zentralvenenkatheter (ZVK)-assoziierte Blutstrominfektionen (CLABSI) sind die häufigste Form von Healthcare-assoziierten infektionen (HAI). Sie sind eine der Hauptursachen für die Morbidität und Mortalität in Krankenhäusern weltweit. Das Auftreten von CLABSI wird durch eine Vielzahl von Risikofaktoren beeinflusst. Daher ist es von entscheidender Bedeutung, Daten über die Entwicklung der Inzidenzraten, der Ursachen und der Antibiotikaresistenzmuster von CLABSI zu sammeln, um empirische Behandlungsschemata zu formulieren und eine robuste Infektionskontrollpolitik zu verfolgen.

**Methode:**

Die prospektive Beobachtungsstudie wurde auf den medizinischen und chirurgischen Intensivstationen für Erwachsene des Mahatma Gandhi Medical College and Hospital in Jaipur von Januar 2023 bis Dezember 2023 durchgeführt. Das CLABSI-Ereignis wurde gemäß der Definition der Centers for Disease Control (CDC) im Rahmen der routinemäßigen Überwachung von HAI ermittelt. Die Inzidenzraten von CLABSI (pro 1.000 Tage mit zentralem Zugang) wurden berechnet. Die Identifizierung und die Empfindlichkeit gegenüber Antibiotika wurde mit einem automatisierten Blutkultursystem durchgeführt.

**Ergebnisse:**

Während des Studienzeitraums kam es bei 59 Patienten zu einer einzelnen CLABSI-Episode mit einer mittleren Prävalenzrate von 15,6 pro 1.000 Zentralvenenkatheter-Tage, wobei die höchste Rate auf der Neuro-Intensivstation (21,6 pro 1.000 Zentralvenenkatheter-Tage) verzeichnet wurde. Das klinisch-demografische Profil zeigte ein Verhältnis von 2,1: 1 zwischen Männern und Frauen, wobei die meisten Fälle bei Patienten im Alter von über 60 Jahren auftraten (25/59; 42,4%). Die häufigste Einstichstelle für den ZVK war die Jugularvene (97%). Die meisten Fälle (88,1%) traten bei Patienten auf, die länger als 10 d auf der Intensivstation lagen. Die Gesamtmortalität lag bei 49,2% (29/59). Der häufigste verursachende Mikroorganismus war *Klebsiella (K.) pneumoniae* (17/59; 29%), gefolgt von *Acinetobacter (A.) baumannii* (14/59; 24%). 83% (33/40) der Gram-negativen Isolate waren gegen Carbapenem resistent, wobei kein Isolat gegen Colistin resistent war.

**Schlussfolgerung:**

Die Studie ergab eine unverhältnismäßig hohe Inzidenz von CLABSI im Vergleich zu Industrie- und Entwicklungsländern, wobei multiresistente Gram-negative Bakterien die vorherrschenden Erreger waren. Die kontinuierliche Überwachung, unterstützt durch Infektionskontrollpraktiken wie Händehygiene und Audits zur Einhaltung von Präventionsbündeln sowie die Einhaltung von Antibiotika Stewardship ist für die Eindämmung von CLABSI entscheidend.

## Introduction

Central venous catheters (CVCs) play a crucial role in intensive care units, serving multiple purposes such as monitoring hemodynamic variables, administering medications, fluids, parenteral nutrition, and blood products, in addition to facilitating blood sampling [1]. These lines include short-term non-tunneled, long-term tunneled, implanted catheters including ports, and peripherally inserted central catheters (PICC); the majority are short-term non-tunneled percutaneous CVCs which are inserted in either subclavian, internal jugular or femoral veins [2]. Given their direct access to the blood stream, these invasive devices can lead to notable complications, most significantly bloodstream infections (BSI) due to invasion of pathogens through CVCs. 

Central-line—associated blood stream infection (CLABSI) is one of the most common healthcare associated infections (HAI) [3]. It is responsible for increased length of hospital stay, increased cost, morbidity and mortality rates up to 12–15% with an odds ratio of in-hospital mortality as high as 2.75 [4]. According to reports of International Nosocomial Infection Control Consortium (INICC), CLABSI rates in intensive care units (ICUs) of developing countries are 5 times higher than the rates in developed countries [5]. In a recent report by the INICC on HAIs, the CLABSI rate was recorded to be 4.55/1,000 central line (CL) days in a consolidated data summary of 45 countries spanning Africa, Asia, Eastern Europe, Latin America, and the Middle East between 2015 and 2020 [6]. 

The incidence rate of CLABSI in low-income countries has been reported in the range of 1.6 to 44.6/1,000 CL days in adult ICUs [7]. However, it is imperative to have authentic data from single centers or hospital settings. Unfortunately, there is a dearth of single-center data from India and limited studies are available on incidence rates of CLABSI. Similarly, the individual hospital settings should also have reliable data on the etiological agents as well as their antimicrobial susceptibility profile, as it varies between developed countries and resource-limited settings [8]. 

Understanding the causative agents and their susceptibility to antimicrobials is paramount for tailoring empirical treatment strategies for HAIs within specific units. This knowledge also aids in monitoring antimicrobial resistance patterns and assessing the efficacy of antibiotic stewardship initiatives. Although country-specific data on incidence rate, etiology and antimicrobial susceptibility are beneficial, it is necessary that individual facilities compile their own data to establish their benchmark and formulate their own empirical regime to obtain a better treatment approach. Thus, the aim of this study was to determine the incidence of CLABSI, identify the causative agents and their antimicrobial resistance profile in ICUs of one tertiary care center in western India.

## Materials and methods

### Study design and settings

This retrospective observational study was conducted in the medical and surgical ICUs of Mahatma Gandhi medical college and hospital, a 1,200-bed tertiary care center in western India from 1^st^ January 2023 to 31^st^ December 2023.

### Inclusion criteria

Patient age >18 years, patients with insertion of temporary non-tunneled CVCs or PICC during the medical and surgical ICU stay or in the department of emergency medicine of our hospital, and patients who developed CLABSI during an ICU stay for more than 48 hours with an indwelling CVC (temporary non-tunneled CVCs or PICC).

### Exclusion criteria

Patients with implanted CVC and ports, patients with any infection incubating at the time of admission to ICU, i.e., samples collected on day 1 of the patient’s stay were blood-culture positive, and patients with secondary BSI.

### Methods

As a part of routine surveillance of HAIs in ICUs, daily rounds were conducted by infection control nurses (ICNs) to record any CLABSI event for patients meeting the inclusion criteria and diagnosed to be a case of CLABSI according to the CDC definition. 

### Case definition

According to CDC-NHSN definitions, CLABSI was defined as a laboratory-confirmed bloodstream infection (LCBSI) 1. if a recognized pathogen was grown in one or more blood cultures after 48 hours of CVC insertion and the pathogen was unrelated to an infection at another location, and 2. if common skin commensals, such as coagulase-negative staphylococci, *Bacillus* species, diphtheroid, micrococci, or *Propionibacterium* species were cultured from two or more blood cultures taken on separate occasions accompanied by at least one of the following clinical features: fever (>38°C) or hypotension [9].

### Incidence rate calculation

The incidence rate of CLABSI was calculated by dividing the total number of CLABSI cases by the number of total central line days multiplied by 1,000. The device utilization ratio for central line was calculated by dividing the number of total numbers of central line days by the total number of patient days.

### Microbiological analysis

Blood samples were processed using an automated blood culture system (BD BACTEC^TM^ FX; Becton Dickinson, Franklin Lakes, NJ, USA ) and incubated at 37°C for five days. Positive blood samples were sub-cultured onto 5% sheep blood agar and incubated on MacConkey agar at 37°C for 24–48 hours; growth was observed the next morning. The identification and antibiotic sensitivity testing (AST) of the isolated microorganisms were carried out using the automated ID/AST system Vitek 2 Compact (bioMérieux SA, Marcy-l’Étoile, France). The quality control strains used for all susceptibility tests were *Staphylococcus aureus* ATCC 25923 and *Escherichia coli* ATCC 25922. The antibiotic susceptibility results were reported in accordance with Clinical and Laboratory Standards Institute (CLSI) guidelines (2023).

### Data collection and statistical analysis

The data included the demographic profile, length of ICU stay, duration of CL, and outcome in terms of discharge, transfer, or mortality of the patients. The data were entered into Microsoft Excel and were statistically analyzed. The categorical variables are presented as percentages. The continuous variables are presented as mean ± standard deviation, and median with interquartile range. 

## Results

A total of 59 patients (single episode) developed CLABSI with a rate of 11.4 per 1,000 central line days during the study duration (Table 1 (Tab. 1)). All CLABSI cases had triple lumens and were non-tunneled. The most common site of CL insertion was the jugular vein (97%), while in the remaining 3%, it was inserted was femorally.

The baseline characteristics profile of patients with CLABSI is shown in Table 2 (Tab. 2). The male:female ratio was found to be 2.1:1. The maximum number of cases were found in the age group >60 years of age (42.4%), followed by the age group of 41–60 years (33.9%), with an overall mean age of 52.5±16.3 years. The minimum and maximum length of stay in ICU was 4 and 52 days, respectively, with a median 19. The majority of cases were patients with longer ICU stays of >10 days (88.1%), with a mean of 21.6±10.7 days. The highest number of cases were associated with prolonged duration of CL (>11 days) in 81.4%, in comparison to <10 days (18.6%). The median number of CL days was 19, with a minimum and maximum duration of 4 and 42 days, respectively. The overall mortality rate was be 49.2% among 59 patients. The mean age of patients who died was 52.2±16.4 years, with an average length of stay of 21.2±10.4 days in ICU. The average duration of CVC catheterization in these patients was 17.9±8.3 days. 

**Figure 1:**
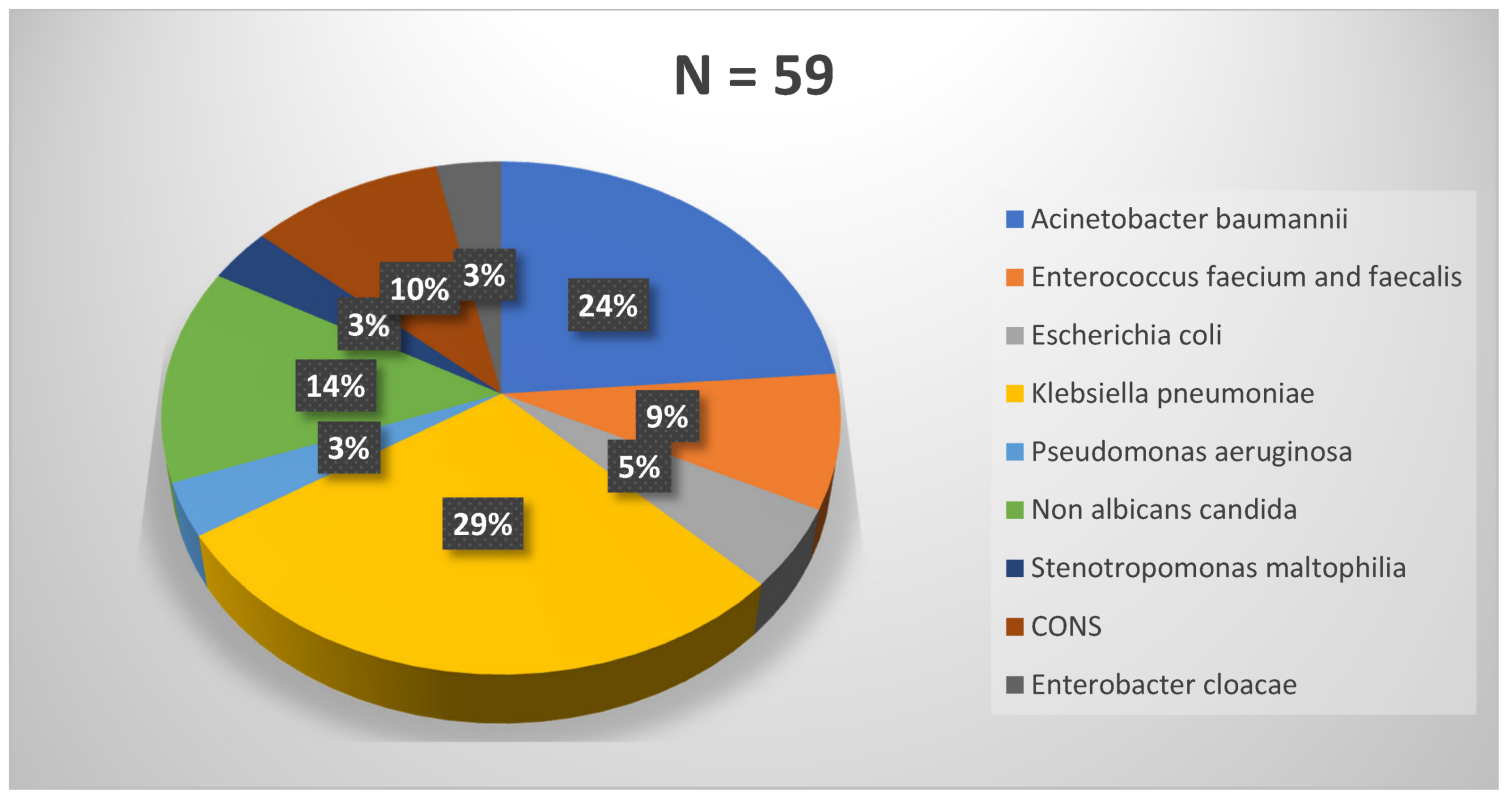
Microbiological etiology of patients with central-line-associated blood stream infections (CLABSI)

Gram-negative bacteria (GNB) were the most common (67.8%) agents followed by Gram-positive cocci (18.6%) and non-albicans *Candida *spp*. *(13.6%). The most common bacterial organisms identified from CLABSI cases were *K. pneumoniae* (29%) followed by *A. baumannii* (24%) and *Candida* spp. (14%) (Figure 1 (Fig. 1)). Among *Candida* spp,. all were non-albicans *Candida*, with *Candida (C.) tropicalis* (75%; 6/8) being most common, followed by *C. famata* (12.5%) and *C. parapsilosis* (12.5%).

Most of the GNBs were carbapenem resistant (33/40; 83%) with the highest susceptibility against colistin (100%), followed by tigecycline (87.5%) and minocycline (72.5%). Out of the 5 *Enterococcus* spp., 40% (2/5) were vancomycin-resistant *Enterococcus* (VRE), while no MRSA was found among the causative pathogens. The rate of methicillin-resistant coagulase-negative *Staphylococci* (MR-CONS) was 67% (4/6). For Gram-positive pathogens, no resistance was found against linezolid. Vancomycin, teicoplanin, and daptomycin each demonstrated 89.5% susceptibility. The non-albicans *Candida* showed 100% susceptibility to echinocandins, amphotericin B, and flucytosine, but 87.5% susceptibility towards fluconazole. 

## Discussion

The present prospective analysis was performed to gather data on CLABSIs in our adult critical care units. Apart from documenting the surveillance data, this information will help assess the current level of infection control practices carried out in the hospital. Furthermore, it will aid in formulation of protocols and practices aimed at reducing the incidence of CLABSI in the future. 

The rate of CLABSI for the year 2023 (Jan–Dec) was found to be 11.4 per 1,000 central line days, with a 0.43 device utilization ratio and 4.9 per 1,000 inpatient days. This is nearly 3 times higher than the rates reported by the INICC for the duration of 2015–2020 in a data summary from 45 countries [6]. Lower incidences from India have been reported by Ganesan et al. [10], Gupta et al. [11], Bammigatti et al. [12] and Kumar et al. [13], ranging from 0.43 to 7.4 per 1,000 central line days. However, comparable and higher incidences reported by single-center studies from different cities of India have been found in the range of 16.4– 47.3 per 1000 central line days [14], [15], [16]. The common reasons for high incidence rates, especially from resource-limited settings, are poor infection control practices including hand hygiene and bundle care, low nurse-to-patient ratio, misuse of invasive devices, patient on femoral line and use of multiple lines, which might occur in our setting as well [4], [14].

Being a tertiary care hospital, our clientele usually conprises referred patients after previous hospitalizations, patients receiving haemodialysis through catheters, oncology and neutropenic patients, which further increases the risk of CLABSI acquisition [17]. Additionally, as it is a medical college, the rapid and constant inflow of medical and nursing students leads to overcrowding in ICUs, along with inconsistencies in training and staff awareness on infection control and bundle care practices. All of this also contributes to higher CLABSI rates. 

In corroboration with our finding of male predominance (68%), various other studies reported the same, with the percentage ranging from 64.9%–72.8% [14], [18], [19], which might be due to males have various risk factors like chronic smoking, alcoholic, tobacco chewer thus also have multiple comorbidities like chronic liver diseases, lung diseases, hypertension thus more chances of developing HAI (CLABSI) in comparison to patients with no or few comorbidities generally female patients.

In the present study, the highest incidence of CLABSI rates were found in the geriatric age group, which agrees with the findings of previous studies [18]. Maqbool et al. [14] highlighted various reasons, such as reduced host defense mechanisms and immunity, underlying comorbidities with increased severity of illness in the elderly, which usually increase the risk of occurrence of CLABSI.

According to the recent 2022 updates by the Society for Healthcare Epidemiology of America (SHEA), the Infectious Diseases Society of America (IDSA) the Association for Professionals in Infection Control and Epidemiology (APIC), the American Hospital Association (AHA), and The Joint Commission, the guideline on strategies to prevent CLABSI in acute-care hospitals updated the various independent risk factors such as use of multi-lumen catheters, prolonged hospitalization before catheterization, prolonged duration of catheter, microbial colonization of insertion site or catheter hub, neutropenia, body mass index >40, prematurity (i.e., early gestational age), reduced nurse-to-patient ratio in the ICU, parenteral nutrition, substandard catheter care (e.g., excessive manipulation of the catheter) and transfusion of blood products (in children) [17]. Among the listed risk factors, not all were monitored in the study, but we nevertheless found increased CLABSI rates associated with diabetes, multiple comorbidities, duration of central line >10 days. However, in all the CLABSI cases, multi-lumen catheters were used, with 97% of them being inserted in the internal jugular vein. In various other studies which analyzed the risk factors, a consistent rise in CLABSI rates with increased device days was unanimously noted [20], [21], [22].

Subha Rao et al. [23] found that all central lines were colonized after 11 days of insertion, while Pitiriga et al. [24] observed a consistent rise in CLABSI rates as the duration of central line use exceeded 10 days, aligning with our results. Thus, in CVC bundle care, daily assessment of the need for a catheter and cleaning the hub whenever used should be regularly emphasized in infection control practices.

Different studies from India and elsewhere have also identified diabetes, immunesuppression, age >60, use of triple-lumen catheters, indication for insertion being total parenteral nutrition, and femoral site for central line insertion as common risk factors [20], [21], [25]. However, Singh et al. [22] did not find any significant association between the femoral site of catheterization and increased CLABSI incidence. The occurrence of CLABSI itself is recognized as a major risk factor for mortality [26]. We found a mortality rate of 49%, which is comparable to the rate documented by a tertiary care center in Mumbai [8]. Lower rates have been noted by various other studies from other developing countries, ranging between 33.3% and 41.9% [26], [27], [28]. Singhal et al. [8] attributed a high mortality rate in their cases to the high prevalence of carbapenem-resistant GNB. 

The data of the past decade from developing countries have shown GNB to be predominant, whereas the literature from developed countries still shows GPC and *Candida* spp. outnumbering GNB as causative agents of CLABSI, as also demonstrated in our study [8], [28], [29]. The present study predominantly found non-albicans *Candida* as a cause of candidemia, which aligns with the results of other studies in Indian ICUs by Singhal et al. [8] and Rudramurthy et al [30]. Consistent with our findings, various studies from developing countries have shown a preponderance of GNB bacteremia (*Klebsiella pneumoniae, Acinetobacter baumannii, Escherichia coli, Pseudomonas aeruginosa*), as reported by Alwazzeh et al. [26] (66%) Al-Khawaja et al. [31] (56%), Singhal et al. [8] (80%). The majority of the GNB isolates in our study were identified as carbapenem resistant, similar to the findings of the surveillance study reported from different parts of India [8], [32]. Rising multidrug-resistant gram-negative bacteria (GNB) pose a significant challenge in the management of these infections. In the present scenario, the ultimate option of empirical therapy in suspected infection in critical care is combination therapy with polymyxins (last resort therapy) as empirical therapy in almost all patients admitted in critical care is alarming due to very high prevalence of CREs in critical care units all over India [10], [11], [14]. All of these demand the enforcement of antimicrobial stewardship programs (AMSP) with strict implementation of antibiotic policy, followed by regular audits conducted by antimicrobial steward team.

MRSA was not isolated in our CLABSI cases, while methicillin-resistant CONS were found in 67%. This is in contrast to a study from the same region which reported 14% MRSA, but no CONS, as the pathogenic organism in CLABSI cases [33]. Fortunately, resistance in Gram-positive isolates is less concerning. However, in India, the prevalence of vancomycin-resistant *Enterococcus* (VRE) is on the rise. Our study identified a 40% rate of VRE. Identifying VRE strains and implementing effective infection control measures are essential to prevent their spread, as increasing resistance in Gram-positive cocci (GPC) would exacerbate the consequences already being faced due to “superbugs”, i.e., multidrug resistant (MDR) GNB [21]. 

### Limitations

One key limitation of this study is that it was conducted at a single institution, potentially limiting the applicability of the results to other healthcare settings in India. Another limitation is that we did not correlate infection control practices, e.g., hand hygiene and CLABSI care-bundle compliance, with the rate of CLABSI events. We intend to include this in future studies.

## Conclusion

In the present study, the incidence of CLABSI was notably higher than that reported in developed countries and even developing countries; the associated pathogens were predominantly MDR GNB. Effective prevention of CLABSI necessitates an understanding of infection rates, potential sources, causative organisms, and their antimicrobial susceptibility patterns. The present study emphasizes the need for continuous and robust surveillance programs to identify the distribution of microorganisms and their resistance profiles in bloodstream infections. Our study also emphasizes the need for a dedicated infection control committee to oversee implementation of care bundles and other infection control practices, along with the formulation of antibiotic policy, all of which is crucial for improving patient care and outcomes.

## Notes

### Authors’ ORCIDs

- Verma P: https://orcid.org/0000-0003-3922-9868
- Gupta S: https://orcid.org/0000-0003-0945-2524

### Funding

None. 

### Acknowledgments

Authors would like to acknowledge the infection-control nurses for their meticulous work of regular surveillance and data collection.

### Competing interests

The authors declare that they have no competing interests.

## Figures and Tables

**Table 1:**
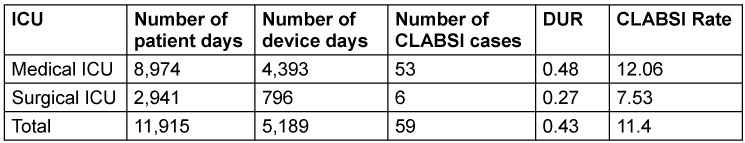
Device utilization rates (DUR) and central-line-associated blood stream infections (CLABSI) rates of medical and surgical ICUs from January 2023 to December 2023

**Table 2:**
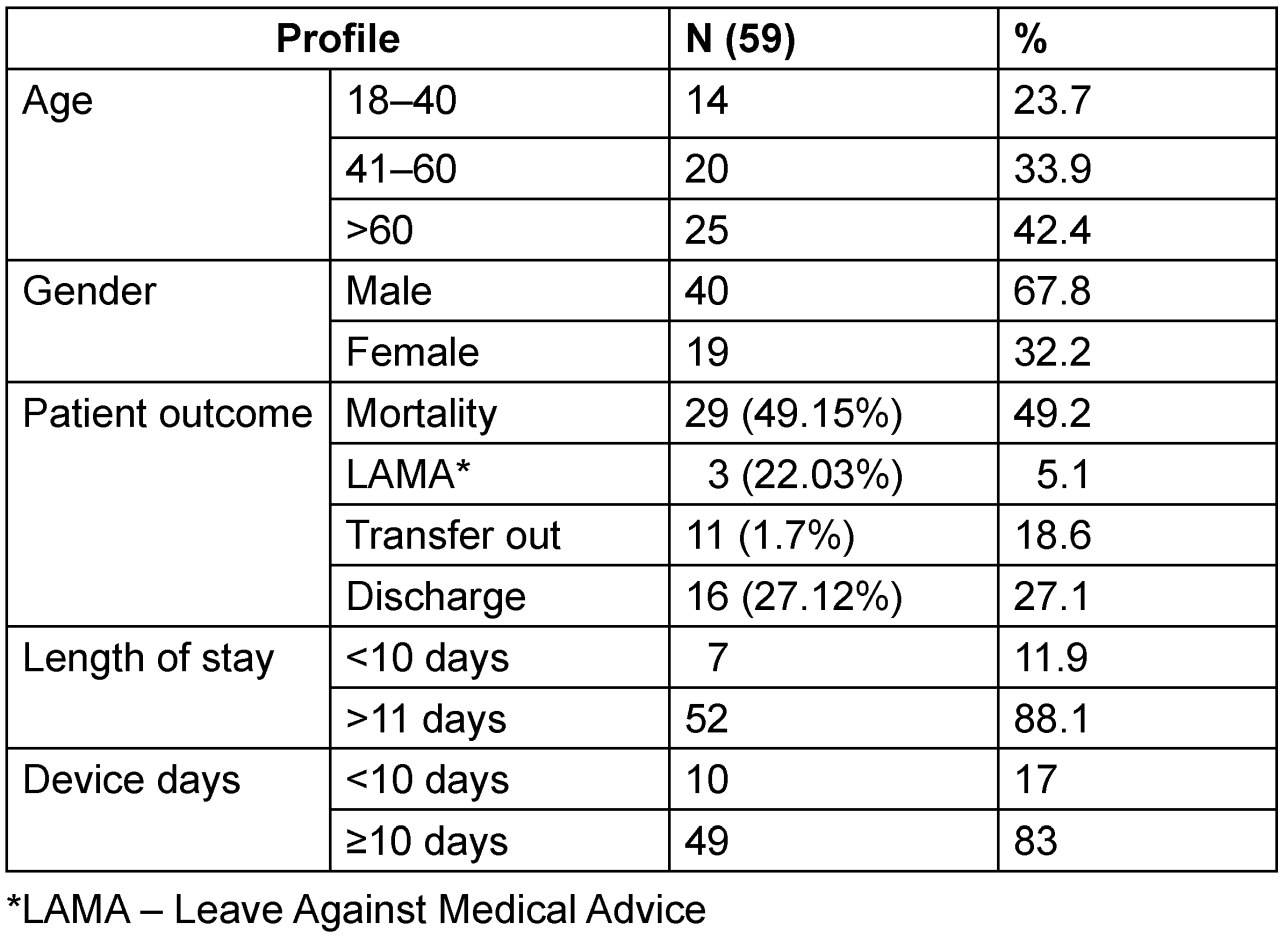
Baseline characteristics of patients with CLABSI (n=59) from January 2023 to December 2023
